# Exercise-induced complete atrioventricular block resulting in cardiac arrest: a case report and review of diagnostic strategies

**DOI:** 10.1093/ehjcr/ytae316

**Published:** 2024-07-02

**Authors:** Fabrício Braga, Tácito Bessa, Matheus Cunha, Roberto Bueno Paiva, Ricardo Stein

**Affiliations:** LPH-Laboratório de Performance Humana, Largo do Ibam, no1–2o floor—Humaitá, Rio de Janeiro, RJ 22271-070, Brazil; Cardiology Departamente, Universidade do Estado do Rio de Janeiro, Rio de Janeiro, RJ, Brazil; Hospital Sírio Libanês, São Paulo, SP, Brazil; Instituto de Cardiologia e Clínica Médica de Joinville, Joinville, PR, Brazil; Hospital Sírio Libanês, São Paulo, SP, Brazil; Aequanimitas Serviços Médicos, Rio de Janeiro, RJ, Brazil; Graduate Program in Cardiology and Cardiovascular Sciences, School of Medicine, Universidade Federal do Rio Grande do Sul, Porto Alegre, RS, Brazil; Internal Medicine Department, Universidade Federal do Rio Grande do Sul, Porto Alegre, RS, Brazil

**Keywords:** Exercise, Arrhythmia, Syncope, Case report

## Abstract

**Background:**

Exercise-induced complete atrioventricular block (EIAVB) is a rare cardiac conduction abnormality presenting challenges in diagnosis due to non-specific symptoms such as exertional dyspnoea, dizziness, and syncope.

**Case summary:**

We present a case of a 76-year-old female with recurrent exercise-associated syncope. Non-invasive exercise testing played a crucial role in diagnosing her condition, revealing EIAVB and underscoring its importance in patients with cardiovascular risk factors.

**Discussion:**

This case provides insight into the pathophysiology of EIAVB, including altered atrioventricular nodal refractoriness and exercise-induced ischaemic imbalances. It highlights the need for heightened clinical vigilance in diagnosing exercise-related syncope, especially in pre-existing cardiovascular conditions. This case underscores the critical importance of non-invasive testing for diagnosing EIAVB, highlighting the necessity of thorough evaluation in patients presenting with ambiguous symptoms and cardiovascular risks. Consequently, it advocates for adherence to guidelines to enhance outcomes and reduce the need for unnecessary invasive procedures.

**Learning points**

- Diagnostic challenges in identifying exercise-induced atrioventricular block (EIAVB) due to its rare occurrence and non-specific symptoms, highlighting the importance of non-invasive exercise testing.
- Understanding the pathophysiology of EIAVB, including altered nodal refractoriness and ischaemic imbalances, emphasizing the need for clinical vigilance and prompt intervention.

## Introduction

While regular physical activity serves as a cornerstone strategy for reducing the risk of various chronic diseases, particularly cardiovascular disorders, intense exercise can unexpectedly induce cardiac rhythm abnormalities, with potentially grave consequences in some cases.^1^ Among these, exercise-induced complete atrioventricular block (EIAVB) emerges as particularly noteworthy due to its rarity.

The customary augmentation of atrioventricular (AV) conduction during exercise, mediated by elevated sympathetic and diminished vagal activity, typically precludes such blockades. Consequently, the emergence of EIAVB during exercise may signify an underlying pathological substrate within the His–Purkinje system (HPS).^2,3^ The non-specific nature of EIAVB symptomatology, encompassing exertional dyspnoea, dizziness, and syncope, poses a significant diagnostic challenge. Non-invasive exercise testing serves as a crucial tool in the identification of exercise-induced syncope and underlying cardiac derangements.

This case report explores a unique presentation of EIAVB in an elderly female patient with a history of recurrent syncope captured during a cardiopulmonary exercise testing (CPET), offering valuable insights into this rare and intricate cardiac phenomenon.

## Summary figure

**Figure:**
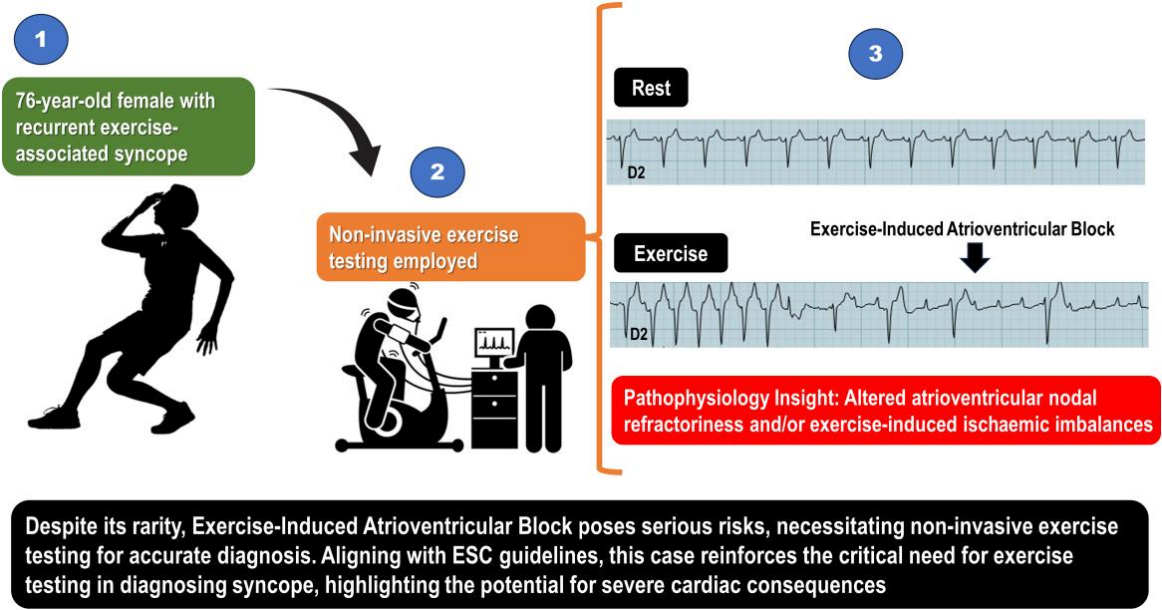


## Case report

A 76-year-old woman was referred for CPET to investigate three episodes of syncope she experienced, with two episodes occurring as she exited the pool during a water aerobics class, highlighting a potential exercise-related trigger. The third episode took place in a hospital while she was visiting her ailing husband. She had no history of syncope during her childhood. She reported high blood pressure and hypercholesterolaemia and regularly took losartan 50 mg/day and rosuvastatin 10 mg/day. Her creatinine and potassium levels were within normal ranges.

**Figure 1:**
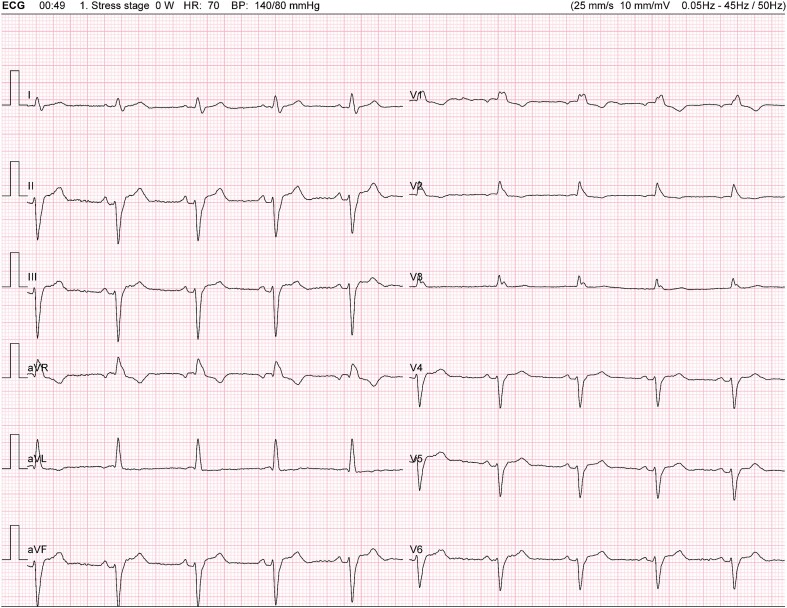
Resting 12-lead electrocardiogram.

Her physical examination revealed no significant abnormalities. A cardiovascular examination showed normal heart sounds with no murmurs. The respiratory examination was unremarkable, with clear breath sounds bilateral. Abdominal examination revealed a soft, non-tender abdomen with no organomegaly or masses. No signs of peripheral oedema or jugular venous distention were observed. Neurological examination was also normal. The resting 12-lead electrocardiogram (ECG) displayed a third-degree right bundle branch block (RBBB) and a left anterior fascicular block (*Figure 1*). The 24-h Holter monitoring showed no pauses, advanced AV block, or any concerning ventricular or supraventricular arrhythmias.

The CPET was conducted using an ergometric bicycle (Lode Corival®, Groningen, Holland) equipped with real-time respiratory gas measurement (MetaSoft Studio®, Cortex®, Leipzig, Germany) and electrocardiographic monitoring (Customed®, Ottobrunn, Germany). The test followed an individualized ramp protocol based on the patient’s maximum predicted capacity, (Wasserman algorithm) comprising 2 min of rest, 3 min of unloaded pedalling, and a subsequent incremental workload phase continuing until exhaustion.

Around the 11th minute of the CPET, she abruptly lost consciousness. She was swiftly taken off the bicycle and laid on the floor, initiating basic life support measures. Approximately 10 s of chest compressions later, she regained consciousness. However, 2 min later, she experienced another loss of consciousness, but this time, she recovered after just 5 s of chest compression. Following this event, she was transferred to the emergency room, where she maintained haemodynamic stability. Cardiac biomarkers, including creatine phosphokinase, troponin I, and brain natriuretic peptide, were measured and found to be within normal ranges.

Upon reviewing the (ECG) records, the following events were documented.

**Figure 2:**
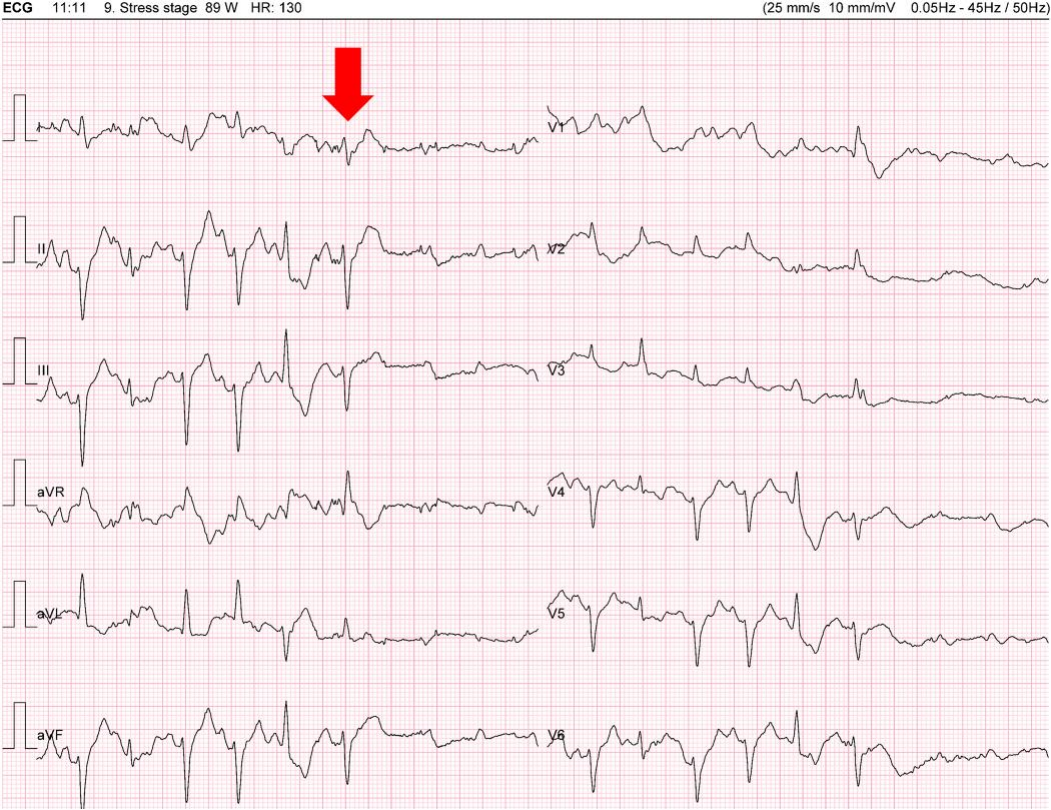
Complete atrioventricular block following a premature ventricular beat (arrow).

**Figure 3:**
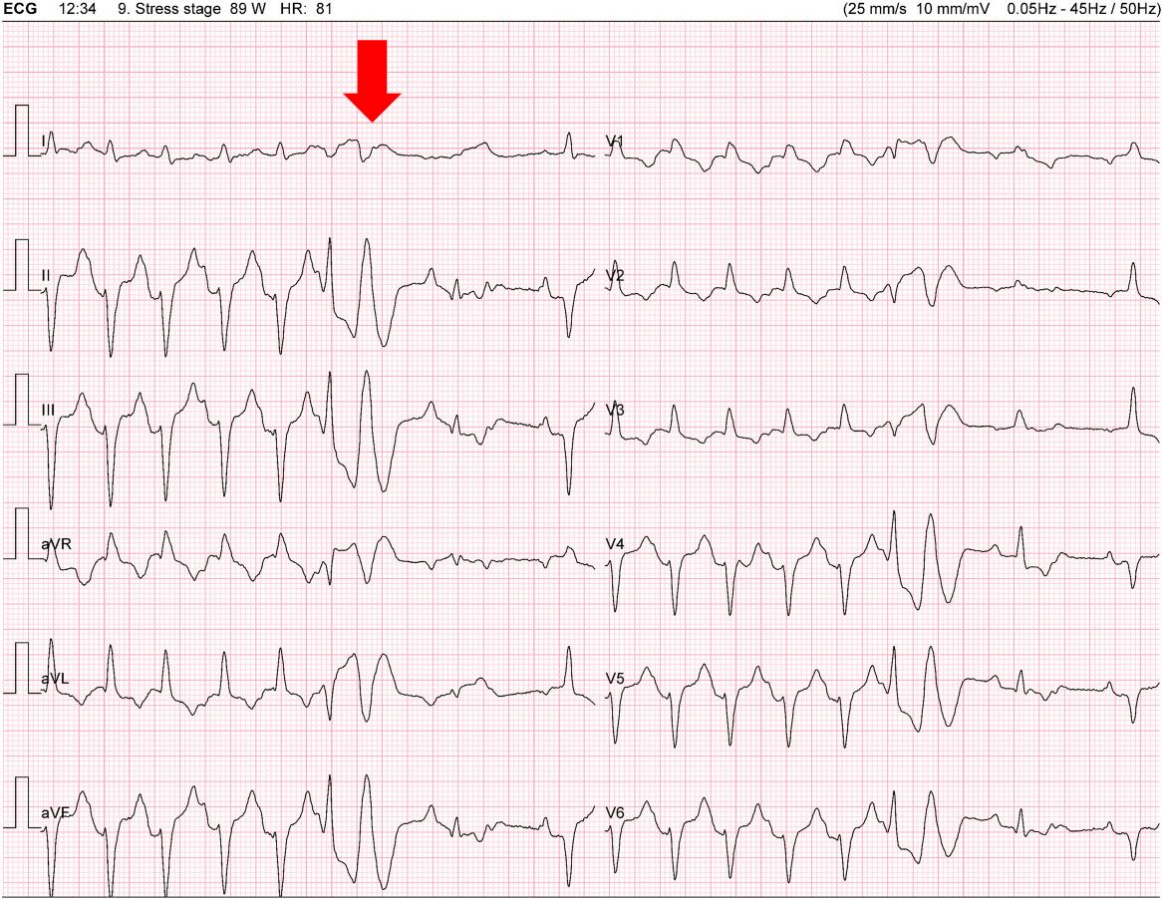
Sinus tachycardia (130 b.p.m.), a 2:1 atrioventricular block after a ventricular couplet (arrow).

**Figure 4:**
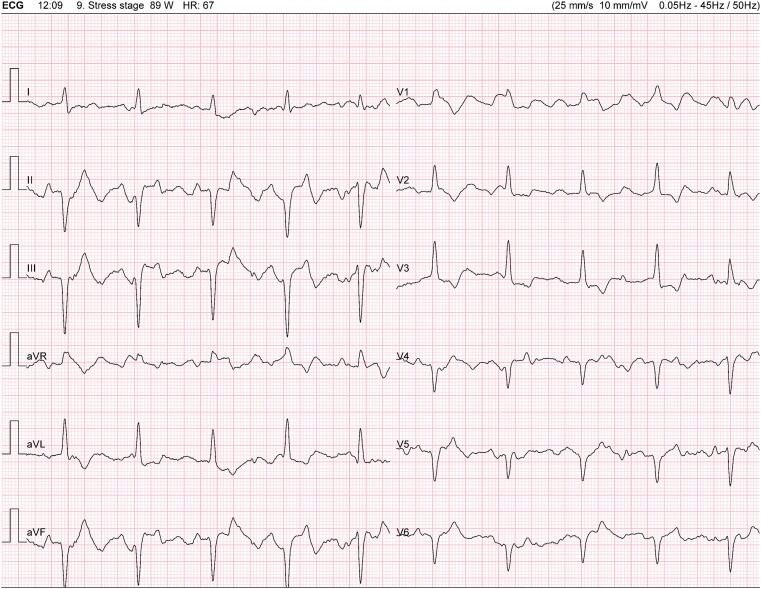
A 2:1 atrioventricular block. Continuation of *Figure 3*.

**Figure 5:**
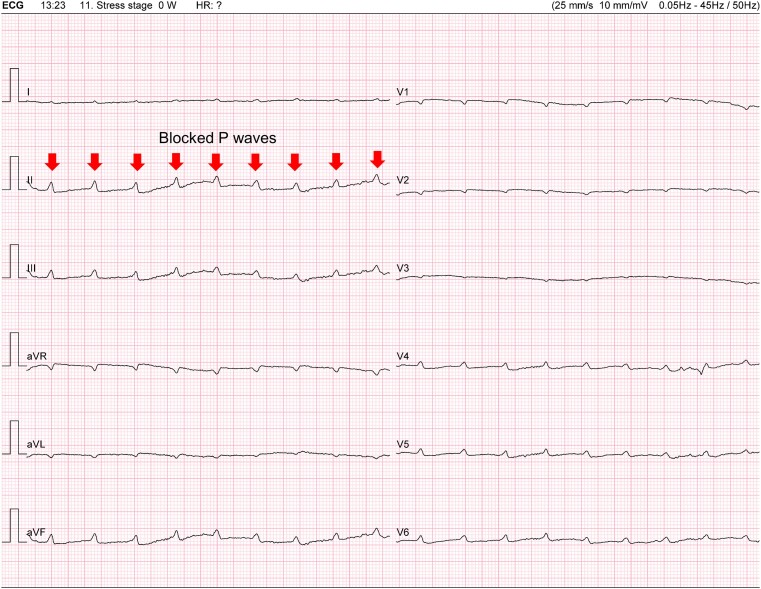
Complete atrioventricular block with a P-wave frequency of 125 b.p.m. P-waves blocked are indicated by a small arrow in lead D2.

- At 11 min and 11 s: complete atrioventricular block (CAVB) following a premature ventricular beat (*Figure 2*).
- At 12 min and 34 s: sinus tachycardia (130 b.p.m.), a 2:1 AV block after a ventricular couplet (*Figures 3* and *4*).
- At 13 min and 23 s: CAVB with a P-wave frequency of 125 b.p.m. (*Figure 5*).

Later, on the same day, she underwent dual-chamber pacemaker implantation. Interestingly, when she experienced CAVB, she had already reached 123 b.p.m., accounting for 85% of her maximum predicted heart rate. Furthermore, her chronotropic index (CI), assessed by the ΔHR/ΔVO_2_ slope, measured 96.2 (normal range: 51–78.4), indicating a very high CI.

At the 3-month follow-up visit, the patient was in good health and had experienced no further episodes of syncope. She continues to be monitored regularly, and as of the writing of this article, she remains alive and well, with no recurrence of syncope.

## Discussion

Physical exertion elicits a spectrum of conduction abnormalities, spanning bundle branch and fascicular blocks to the rare and severe manifestation of CAVB.^4–6^ Pre-existing cardiac pathologies may precipitate CAVB during exercise stress, leading to syncope, though this remains uncommon. A comprehensive literature review emphasizes the scarcity of EIAVB reported during exercise testing, highlighting the exceptional nature of this heart block.

The underlying mechanisms of EIAVB remain enigmatic, with two primary hypotheses emerging. The first posits alterations in the AV node refractory period and a compromised conduction system, while the second suggests exercise-induced AV node ischaemia due to an imbalance in oxygen supply and demand. Our case, involving an elderly woman with established risk factors for ischaemic heart disease, underscores the importance of considering such underlying conditions in patients presenting with exercise-induced syncope^7,8^

Boran *et al*.,^9^ encompassing 2200 patients, revealed a meagre 0.45% (10 individuals) exhibiting exercise stress test-induced ischaemia and conduction anomalies. These included four cases of left anterior fascicular block, two with left posterior fascicular block, and four with RBBB. Notably, no instances of CAVB were documented in this large-scale study. In contrast, our case features a patient for whom the invasive coronary angiogram (ICA) conclusively negated the presence of significant obstructive coronary disease prior to pacemaker implantation, underscoring the uniqueness of this case. The ICA disclosed a 30% stenosis in the proximal segment of the left anterior descending artery and a 40% stenosis in the mid-segment of the right coronary artery, clearly demonstrating the absence of obstructive coronary artery disease. These findings highlight the distinctive nature of the patient’s cardiac condition.

Exercise testing (in this case a CPET) facilitated the determination of our patient’s CI, unveiling a crucial aspect in the context of CAVB. With a CI significantly exceeding the normal range, this case aligns with Sirichana *et al*.^10^ findings associating high CI with cardiovascular impairment. Intriguingly, the elevated CI in our patient coincided with CAVB, deviating from the expected enhanced AV conduction associated with a strong sympathetic response. This underscores the intricate nature of cardiac electrophysiology in exercise-induced arrhythmias and emphasizes the need for individualized patient evaluations.

Real-time ECG monitoring is essential for diagnosing EIAVB, necessitating immediate cessation of the test and intervention, even in asymptomatic cases. Determining the specific nature and location of the AV block, whether supra- or infra-Hisian, remains challenging. In situations where ischaemic heart disease is excluded, degenerative diseases like Lenegre’s or Lev’s become likely causes for advanced heart blocks.^11^ To distinguish between supra-Hisian (located above the bundle of His) and infra-Hisian AV blocks during exercise testing, it is critical to note that supra-Hisian blocks typically show improvement due to increased sympathetic activity, which accelerates AV nodal conduction. On the contrary, infra-Hisian blocks demonstrate minimal to no response to exercise, indicating a lack of improvement with sympathetic stimulation.^12^ This insight is vital, as infra-Hisian blocks may necessitate pacemaker implantation due to the high risk of progression to CAVB and potential sudden death. Electrophysiological studies might be necessary to investigate HPS abnormalities, especially in high-risk patients or when non-invasive tests like CPET do not conclusively determine the AV block’s level.^13^

Our approach, in line with ESC pacing and syncope guidelines,^14,15^ emphasizes using non-invasive imaging and exercise tests to assess structural heart disease and left ventricular function before considering pacemaker implantation. This method aligns with guideline recommendations for evaluating patients with symptomatic bradycardia through cardiac imaging to identify structural heart conditions and check left ventricular systolic function, particularly in patients under 60 suspected of having specific pathologies that may require a pacemaker.

Moreover, the case shows a cautious approach, opting for detailed non-invasive evaluation over more invasive options like implantable loop recorder implantation, to avoid unnecessary procedures. By adhering to these guidelines and incorporating current recommendations into our diagnostic strategy, we aim to highlight the critical balance between thorough patient evaluation and the prudent use of technology to avoid unnecessary invasive procedures.

## Conclusion

In conclusion, our case underscores the critical importance of adhering to the current guidelines to circumvent unnecessary invasive procedures. Our findings demonstrate that even in rare conditions like EIAVB, a thorough guideline-driven approach can lead to accurate diagnoses and significantly benefit patients. This methodology not only ensures optimal patient care but also highlights the transformative impact of guideline adherence on clinical outcomes, fostering a more precise and cautious application of medical interventions.

## Lead author biography


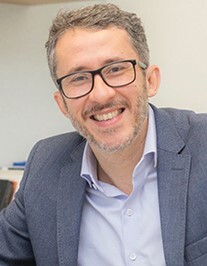


Fabrício Braga is a cardiology PhD candidate at Universidade Estadual do Rio de Janeiro, Brazil, and holds a master’s degree in cardiology from Universidade Federal do Rio de Janeiro, Brazil. He is a specialist in cardiopulmonary testing and a fellow of the European Society of Cardiology since 2022. Braga serves as the Medical Director of LPH and Chief Medical Officer of Triathlon Brasil. His research focuses on sports science, cardiac rehabilitation, exercise physiology, and the effects of COVID-19 on physical fitness in athletes and non-athletes, alongside studies on the health benefits of physical exercise.

## Data Availability

The data underlying this article will be shared on reasonable request to the corresponding author.

## Notes

**Consent:** This case report subject provided informed consent following comprehensive discussions regarding the case details, in line with COPE guidance. Obtaining informed consent upholds ethical standards, safeguards the patient’s autonomy, and ensures confidentiality.

**Funding:** None declared.
